# Multi-Year Study of the Chemical and Sensory Effects of Microwave-Assisted Extraction of Musts and Stems in Cabernet Sauvignon, Merlot and Syrah Wines from the Central Coast of California

**DOI:** 10.3390/molecules27041270

**Published:** 2022-02-14

**Authors:** L. Federico Casassa, Paul A. Gannett, Nicholas B. Steele, Robert Huff

**Affiliations:** 1 Wine and Viticulture Department, California Polytechnic State University, San Luis Obispo, One Grand Ave., San Luis Obispo, CA 93407, USA; pgannett@calpoly.edu (P.A.G.); nbsteele@comcast.net (N.B.S.); robertjhuff33@gmail.com (R.H.); 2 Jonata Winery, P.O. Box 191, Buellton, CA 93427, USA; 3 Valkyrie Selections, LLC, Healdsburg, CA 95448, USA

**Keywords:** phenolic compounds, anthocyanins, tannins, wine color, microwave, stems, sensory analysis

## Abstract

Microwave technology (MW) was applied to musts and stems over three consecutive vintages in Cabernet Sauvignon, Merlot and Syrah wines from California (USA). Stems were added to musts at a rate of 50 and 100% (50% Stems and 100% Stems), either as untreated or after MW (50% MW Stems and 100% MW Stems). Stem additions lowered ethanol (up to 1.15% *v*/*v* reduction), but increased pH (up to 0.16 units) and the tannin content of the wines. In 2016, tannins increased by 103% (100% Stems), and 124% (100% MW Stems). In 2017, tannins increased by 39% in stem-added Merlot wines and by 63% (100% Stems) and 85% (100% MW Stems) in Syrah wines. In 2018, tannins in Syrah wines increased by 250% (100% MW Stems) and by 743% (100% Stems). Wines made with 50% Stems exhibited intermediate tannin contents. Must MW increased flavonols (up to 278% in Syrah wines), monoglucosylated, acylated and anthocyanin-derived pigments. Stem additions reduced wine color and polymeric pigment formation in Syrah. Must MW decreased the perception of coarseness and herbaceous flavors in Merlot, whereas stem additions increased herbaceous aromas in Syrah. Despite higher tannin contents in stem-added wines, no concomitant increases in astringency were observed.

## 1. Introduction

The time-honored motto that “*wine is made in the vineyard*” is widely accepted by the wine industry and empirical as well as scientific evidence appears to support this view. The statement implies that the chemical composition of the grapes (and the resulting wines) is determined by the interactions between the grape cultivar (cv.), the vineyard site, the vintage, and the applied viticultural practices. This view does not exclude the contribution of enological treatments known to affect the chemical and sensory profiles of the wines during winemaking and subsequent stages. In fact, some studies have reported a larger impact of winemaking over viticultural practices [1]. Indeed, several practices applied during winemaking of red grapes can alter the biochemical, chemical, and sensory composition of the resulting wines. Phenolic compounds are key component of grape and wine chemical composition. They appear in grapes and wines as a rather heterogenous family of highly bioactive compounds including light-absorbing molecules such as anthocyanins and flavonols, and others able to precipitate with proteins such as tannins. Phenolic compounds in grape berries are minor components relative to sugars and acids, with tannins, anthocyanins and flavonols accounting for up to 7.5 mg/berry, 14.7 mg/berry and 6 mg/berry, respectively [2], whereas they range from 300 to 3000 mg/L in grape juices [3]. In finished wines, phenolic compounds rarely exceed the threshold of 4000 mg/L (thus less than 0.4% by weight in finished wines). Yet phenolic compounds are key determinants of color, taste (bitterness) and tactile sensations such as mouthfeel and astringency of red wines. In broad terms, the most relevant phenolic compounds in red wines include anthocyanins and their derivatives, responsible for wine color; flavonols, that drive copigmentation reactions; and tannins, responsible for mouthfeel properties such as astringency. Polymeric pigments, an heterogenous family of anthocyanin–tannin reaction products are typically formed during the winemaking process [4]. 

In the past 25 years, a host of novel winemaking techniques aimed at increasing the extraction and retention of phenolic compounds in the finished wines have emerged within the winemaking community, thus subsequently drawing scientific scrutiny. An underlying prerequisite is for these technologies to be environmentally sustainable, limit water, energy usage, and the addition of exogenous chemical compounds, and to avoid the generation of harmful environmental byproducts. Implied is the requirement that these technologies be scalable to industrial (winery) conditions. Moreover, they should also be applicable to winemaking byproducts such as red and white wine pomace (skin and seeds recovered after pressing or after the fermentation process) and yeast lees. Equally important, these approaches must be financially and logistically affordable vis à vis traditional practices. 

Among these novel technologies, ultrasound-assisted extraction (UAE) and microwave-assisted extraction appear to be good candidates that conform to the prerequisites outlined above. Ultrasound-assisted extraction of grape pomace, whose principle hinges upon facilitation of mass transfer and solvent penetration processes, as well as cavitational collapse of grape cells, was particularly efficient at extracting phenolics from pomace when combined with supercritical CO_2_ extraction [5], but it yielded variable results depending upon the specific composition of the grape cv. in Primitivo (positive effect), Nero di Troia (no effect) and Aglianico (positive effect) [6].

The industrial application of microwave treatment of numerous food products dates to 1964, with early applications involving industrial-scale heating (blanching, thawing, and drying) [7]. Microwave-assisted extraction (MW) depend on the ability of water molecules to align with the direction of a fast-oscillating electric field created by the MW process, which produces internal friction and thus volumetric heating [8]. Within the domain of novel winemaking technologies, the MW process is ecologically friendly, as it does not require water input nor any chemical aids and does not generate any byproducts [9]. A study reported that MW applied to Pinot noir musts produced a four-fold increase in tannin concentration, along with a decrease in the native grape yeast-derived populations [10]. In wines produced from Merlot grapes harvested at three different maturity levels, application of MW to musts prior to alcoholic fermentation led to initial early increases in wine color (from 175% to 300%), but only a 52% improvement in wine color was retained in the wines made from unripe grapes after 150 days post-crushing [11]. In wines from cv. Dornfelder, MW applied at 1200 W (approx. 400 W/kg), to attain 80 °C, significantly increased the extraction of total phenolics and the antioxidant capacity relative to the effect of a thermomaceration treatment. However, no effect on color (CIELab parameters) was observed in the finished wines after maturation [12]. Mechanistically, MW may enhance extraction of phenolics by increasing mass transfer processes such as diffusion [11]. As recently shown in Cabernet Sauvignon, the application of MW may indirectly result in enhanced phenolic extraction at the early stages of winemaking by decreasing the activity of polyphenol-oxidases through partial enzyme denaturation [13]. Lastly, applied to wines during aging, both MW and UAE have shown promising results [14], suggesting these technologies may be versatile enough to also treat wines at the beginning or during the aging process.

The increased demand for wines with distinctive aromas and textures has led winemakers to explore the inclusion during maceration of other vine components, including leaves [15], vine shots [16] or, more commonly, grape stems. Although stem inclusion using whole clusters has been traditionally used in Pinot noir winemaking under non-irrigated conditions in Burgundy (France), stems have also been regarded as potentially imparting negative sensory characteristic such as herbaceous or vegetal aromas [15,17,18]. This is because under irrigated viticulture, stems are rarely lignified and remain green at harvest time. However, stems represent a rich and virtually cost-free source of phenolic compounds (vis à vis exogenous tannin additions), with a reported total phenolic content that can be as high as 38,400 mg/100 g of dry mass [17]. Thus, techniques that minimize the risk of extraction of herbaceous flavors into wine while allowing the extraction of desirable phenolics and other aroma compounds from added stems have been developed. For example, a study conducted with Pinot noir at the commercial winery scale in which stems were dried outdoors and added prior to alcoholic fermentation reported that these dried stems increased tannins by 90% and 137% over two vintages, while resulting in wines with enhanced herbal, fruity, and astringent sensory characteristics [19]. In another report also in Pinot noir, stems were pretreated with MW (52 °C, 5 min), prior to addition to the fermentation of three different clones of this cv. Relative to Control wines, MW Stem wines exhibited an 8-fold, 19-fold and 13-fold increase in wine tannins for clones 2A, 115 and 777, respectively. As well, the concentration of polymeric pigments was doubled, and attributed to the extraction of stem-derived tannins [20].

Herein, we report the results of a multi-year study involving three consecutive vintages (2016, 2017 and 2018), in the Central Coast of California, whereby Cabernet Sauvignon, Merlot and Syrah grapes were processed by treating musts and stems with MW prior to alcoholic fermentation. We report a comprehensive chemical and chromatic characterization of the resulting wines at the stages of maceration, and after extended bottle aging to determine over these three vintages the short- and long-term chemical and chromatic effects of MW technology in the resulting wines. Moreover, sensory analysis was applied to characterize Merlot and Syrah wines from the 2016 vintage. 

## 2. Results and Discussion

In this multi-year study spanning three consecutive vintages, the effect of microwave technology (MW) applied to stems and musts prior to alcoholic fermentation was studied in three different cvs., Cabernet Sauvignon, Merlot and Syrah grown in the Central Coast of California. The MW technique was applied to all of the individual musts, as well to a variable portion of the stems originally present in the grapes, ranging from 100% (2016 and 2017 vintages) to 50% and 100% (2018 vintage) in triplicate fermentations. Various aspects, including the basic chemical composition of the fruit at harvest time, the basic chemical composition of the resulting wines, the extraction of phenolics and their evolution during bottle aging, along with their chromatic characteristics, and some selected aspects of the sensory composition for the 2016 Syrah and Merlot wines, were considered. 

### 2.1. Basic Chemical Composition of the Grapes

Table 1 shows the basic chemical composition of the grapes during the three vintages studied, along with aspects related to the vintage, that is, the accumulation of growing degree days (GDD) in each vineyard site. The percentage of stems calculated based on fresh weight (FW) is also shown. Crucially, the source of the fruit was kept constant for all the three vintages to determine any potential contribution of vintage on the final results. The vintage effect was more pronounced during the 2017 season, which was a much warmer vintage in California in comparison to 2016 and 2018 (Table 1). 

As expected, there were large differences in the basic chemistry of the grapes at harvest as a function of each cv. Sugar levels at harvest measured as Brix were higher in Syrah fruit, whereas pH values were higher in Merlot berries, and concomitantly, titratable acidity (TA) and L-malic acid levels were also comparatively lower in Merlot fruit. Potassium levels were generally higher in Merlot fruit as well, possibly contributing to the lower TA and higher pH values observed in Merlot fruit [21]. 

Cabernet Sauvignon fruit showed the highest percentage of stems relative to Merlot and Syrah, whereas Merlot showed the lowest percentage of stems at harvest time. Syrah showed an intermediate stem percentage relative to Cabernet Sauvignon and Merlot fruit.

### 2.2. Basic Chemical Composition of the Wines

Table 2, Table 3 and Table 4 present the basic chemical composition of the wines of the 2016, 2017 and 2018 vintages post-bottling, respectively. A combination of one-way ANOVA, within each cv., as well as two-way ANOVA including all the three cvs. tested for each vintage were conducted to visualize the separate effects of the cv. and the winemaking techniques on the various aspects of the wine’s basic chemical composition. 

All the wines completed both alcoholic and MLF, and levels of acetic acid were well below the legal limit. Consequently, parameters such as glucose + fructose, acetic, malic and lactic acid levels are not further discussed here.

In 2016, ethanol levels decreased consistently in all the three cvs. produced by addition of stems, with or without MW. A two-way ANOVA confirmed this trend, showing an overall ethanol reduction of 0.5% *v*/*v* in the 100% Stem wines relative to Control wines (Table 2). Because stems are predominantly composed of water, which accounts for 80% of stem fresh weight [17], this reduction in ethanol content may be attributed to the diluting effect of water from the stems, as previously shown elsewhere [17,20]. Notably, a reduction in the ethanol content in finished wines is nowadays a desirable trait to be achieved during winemaking. For example, lower ethanol levels achieved through the use of *non-Saccharomyces* yeast cultures are currently associated with more balanced wines, and enhanced perception of freshness [22]. Herein, reductions in ethanol were instead achieved by stem addition, suggesting that this practice may be used as a tool to lower ethanol content in warm climates. Overall, pH values increased in the stem-added wines by an average of 0.09 units in the 100% Stems wines and by 0.13 units in 100% MW Stems wines (Table 2). Because potassium represents the main mineral element in stems (accounting for 0.9% of total ash content) [17], these increases in pH can be attributed to the extraction of potassium from the stems and may ultimately have negative consequences for the wines such as lowering the efficiency of SO_2_ additions and resulting in a faster evolution of wine color during aging due to higher pH.

A similar trend was observed in the 2017 wines, wherein the application of the 100% Stem and 100% MW Stems treatments decreased ethanol levels but increased pH. For example, relative to Control wines, an ethanol reduction of 1.15% *v*/*v* was realized in the 100% Stem wines whereas pH concomitantly increased by 0.08 units (Table 3).

In the 2018 vintage, the application of the Must MW treatment was discontinued and instead different percentages of stems, namely 50% and 100% were tested. As previously observed in the wines of the 2016 and 2017 vintages, stem additions led to reductions in ethanol and increases in pH in comparable magnitude to those observed in the two previous vintages. For example, a two-way ANOVA showed that the wines of the 100% MW Stems treatment had an ethanol content 0.81% *v*/*v* lower than that of Control wines, whereas the pH resulted in 0.16 units higher in these wines (Table 4).

Overall, most parameters pertaining to the basic chemical composition of the wines were unaffected by the winemaking techniques herein essayed, with two main prominent exceptions. That is, stem additions led to potentially sensory-relevant reductions in ethanol content and increases in wine pH. Whereas achieving lower ethanol content has positive sensory implications, as discussed above, higher pH values may be deemed negative vis à vis the microbial stability and chemical evolution of the resulting wines during aging.

### 2.3. Detailed Phenolic and Chromatic Composition of the Wines during Winemaking and Bottle Aging

Phenolic compounds, including anthocyanins, tannins and flavonols, are key components of the wine’s chemical matrix, modulating key sensory features such as color, tactile sensations (e.g., astringency) and copigmentation reactions [23]. Moreover, winemaking artifacts such as polymeric pigments, which form progressively throughout winemaking and bottle aging, are known to impart both mouthfeel characteristics and color stability throughout bottle aging [24,25]. To assess the immediate as well as long-term effect of the MW-based techniques herein studied, specific phenolic classes known to impart critical sensory characteristics were followed throughout winemaking and bottle aging. Specific chromatic characteristics, such as wine color intensity as well as CIELab color panels showing the actual visual aspect of the wines, including the CIELab color difference (ΔE*) were also recorded to fully capture the effect of these techniques on the wine’s color characteristics.

#### 2.3.1. 2016 Vintage Wine

**Figure 1:**
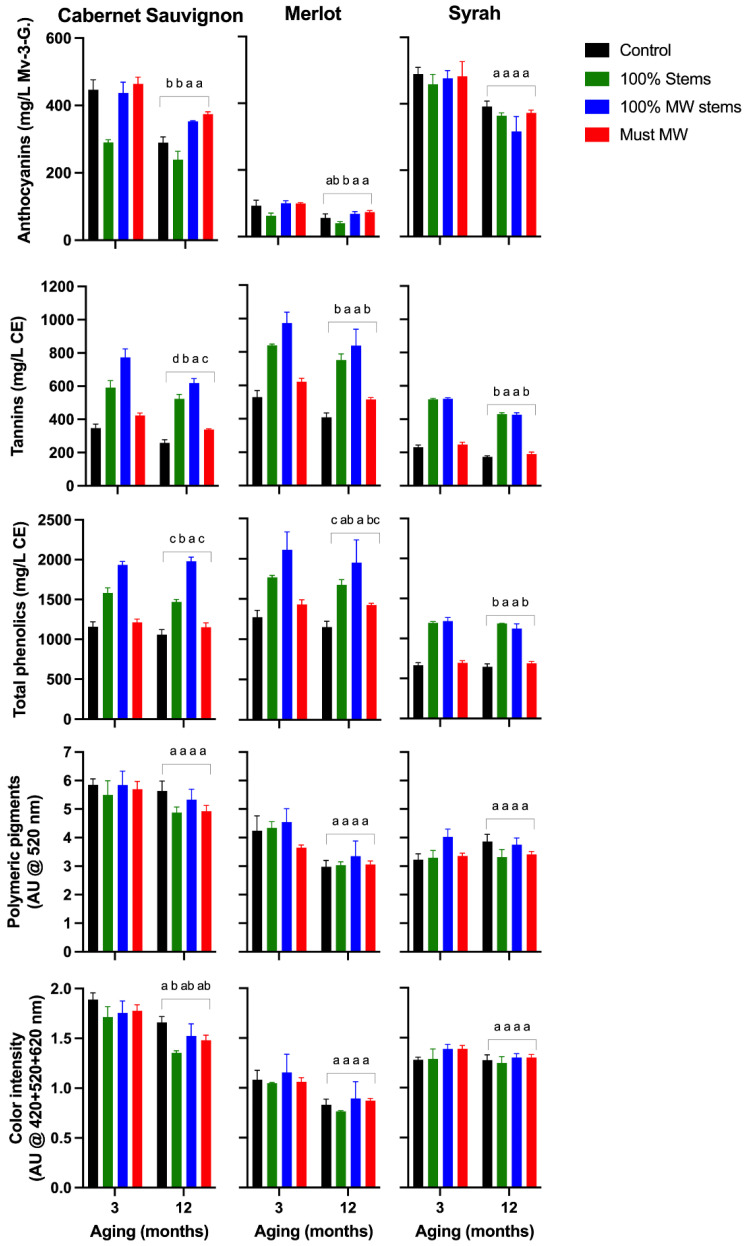
Evolution during bottle aging of selected phenolic classes and wine color in the wines of the 2016 vintage. Different letters in the last sampling point (12 months of bottle aging), indicate significant differences for Fisher’s LSD test and *p* < 0.05. Mv-3-G.: malvidin-3-glucoside equivalents; AU: absorbance units; CE: catechin-equivalents.

**Figure 2:**
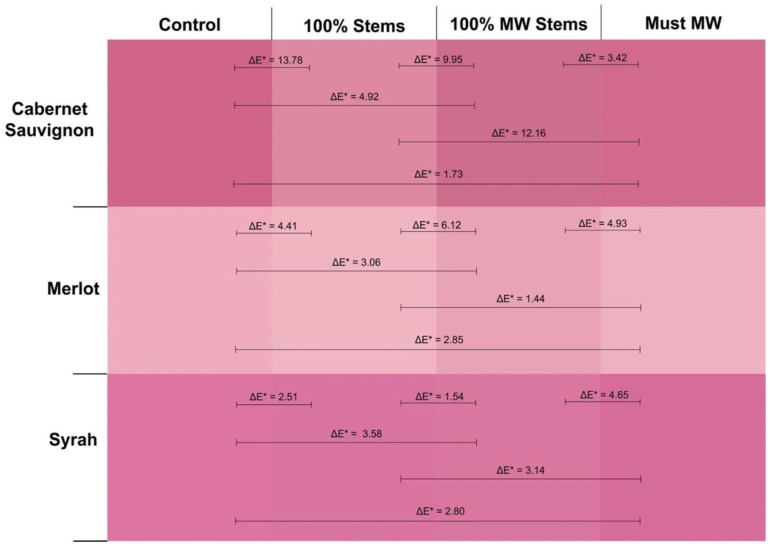
Visual depiction of the actual color of the 2016 wines of the three cvs. and four winemaking treatments as seen through a 1 mm path length quartz cuvette at the time of pressing (day 12). ΔE* values are shown between each pair of treatments for a given cv.

The wines of the 2016 vintage were evaluated after 3 and 12 months of bottle aging (Figure 1), whereas the chromatic characteristics, visual aspect, and the CIELab color difference between the wines of the different winemaking treatments were measured at pressing (Figure 2). The individual effect of cv. as well as the aggregate effect of each winemaking technique is provided in Supplemental Table S1.

Anthocyanins were generally higher in Cabernet Sauvignon and Syrah relative to Merlot wines, which showed anthocyanin levels somewhat below average of what is normally recorded in the wines of this cv. [11]. Whereas none of the winemaking techniques affected the anthocyanin content of Syrah wines, 100% MW Stems and Must MW wines improved the anthocyanin content of Cabernet Sauvignon wines (Figure 1). 

Tannins levels were comparatively higher in Merlot wines and, in general, addition of stems with or without MW treatment, and irrespective of the cv., caused a sharp increase in the tannin content of the resulting wines. For all the three cvs. considered, tannin increased by 103% and 124% in 100% Stem wines and in 100% MW Stem wines relative to Control wines (Supplemental Table S1). This noticeable increase in wine tannin content upon stem addition can be attributed to the contribution of stem-derived tannins. Tannins are possibly the most abundant phenolic class in stems [17], thus adding them to wines boost tannin extraction. In good agreement with the results presented here, a study reported that additions of 100% dried stems in Pinot noir increased tannins in the resulting wines by 90% and 137% over two consecutive vintages relative to Control wines [19]. In the present study, the application of MW to the stems led to an additional tannin extraction relative to that observed when only fresh stems were added pre-fermentation, possibly due to the extractive effect of MW on the cellular structure and integrity of the stems.

The concentration of total phenolics generally mirrored and followed the trend previously observed for tannins. That is, significantly higher levels of total phenolics were observed in the wines of the 100% Stem and 100% MW Stem treatments, irrespective of the cv. (Figure 1 and Supplemental Table S1).

Although the content and formation of polymeric pigments is dependent on the anthocyanin and tannin content of the wines, among other factors, no effect on the initial (3 months post-bottling) or long-term content and formation (12 months post-bottling) of these pigments was observed in the 2016 wines. This finding deserves discussion as in the present study additions of stems significantly altered the tannin to anthocyanin ratio of the resulting wines. This altered ratio, however, did not significantly impacted the formation of these desirable polymeric pigments. Despite being approximately 200 mg/L higher in tannin than their Cabernet Sauvignon and Syrah counterparts, the tannin content of Merlot wines did not result in more, but rather comparatively lower polymeric pigment content (Figure 1). Overall, these results suggest that polymeric pigment formation is not solely regulated by anthocyanins and tannins but by other components of the wine matrix, in addition to the well-understood effect of external factors such as temperature and oxygen during storage. Possible regulators of polymeric pigment formation include wine pH [26], SO_2_, the proportion of anthocyanins in the positively charged flavylium cation [27], and the presence of other macromolecules such as polysaccharides [28]. The latter may even be part of the macromolecular structure of polymeric pigments [29]. 

In the case of wine color intensity (Figure 1), minor effects were observed, albeit a slight trend favoring Control wines was uncovered by a two-way ANOVA (Supplemental Table S1).

Figure 2 displays the actual visual aspect of the 2016 wines at pressing as seen through a 1 mm path length quartz cuvette. In addition, ΔE* values between any given pair of wines are also shown. This parameter, known as CIELab color difference predicts whether a chromatic difference between two wines will result in a perceptible visual difference under CIELab standard conditions (10° standard observer and the illuminant D65). When this difference is >2.8 CIELab units, chromatic differences between the two wines are expected to be discernible by the human eye [30]. As seen in Figure 2, this analysis captured differences that were not evident by the simple addition of discrete wavelengths such as in the case of wine color (AU 420 + 520 + 620 nm). For Cabernet Sauvignon wines, Control wines had higher color saturation than 100% Stem and 100% MW Stem wines, with ΔE* values well above the threshold of 2.8 CIELab units, which indicates that these differences would be readily perceivable by the human eye. The addition of 100% Stems was especially detrimental to wine color intensity, although treatment of stems with MW seemed to curb this color loss. Conversely, the Must MW wines were in turn more saturated than those made by stem additions at pressing, but no different from Control wines (ΔE* = 1.73, Figure 2, top panel). 

The 2016 Merlot wines showed lower color saturation than the Cabernet Sauvignon and Syrah wines of this vintage (Figure 2, middle panel). The highest perceived color, however, was observed in the wines of the 100% MW Stem treatment. ΔE* values were above 2.7 CIELab units in all comparisons contrasting MW Stems wines with the other treatments. This implies that these differences were perceivable by the human eye. The highest color difference was recorded when 100% MW Stems wines were chromatically contrasted against the 100% Stem wines. This suggests that for Merlot, MW applied to the stems prior to their addition to the fermenters promoted higher color saturation. This increase in color saturation cannot be attributed to polymeric pigments as they did not differ between 100% Stem and 100% MW Stem wines (Figure 2). It may be instead ascribed to differences in anthocyanins and tannins that favor 100% MW Stem wines. A higher concentration of anthocyanins in these wines could increase color saturation as anthocyanins have a higher molar extinction coefficient than that of polymeric pigments [20]. The significantly higher total phenolic content of 100% MW Stem wines (though not relative to 100% Stem wines) may implicate other phenolic classes in these differences, as well. 

The opposite trend was found for Syrah, where lower color saturation was observed in the 100% MW Stem wines (Figure 2, bottom panel), along with the 100% Stem wines. These wines were generally distinguishable with lower color saturation than their Control wine counterparts. At 12 months of bottle aging, however, these differences were no longer evident (Figure 1 and Supplemental Table S1).

#### 2.3.2. 2017 Vintage Wines

**Figure 3:**
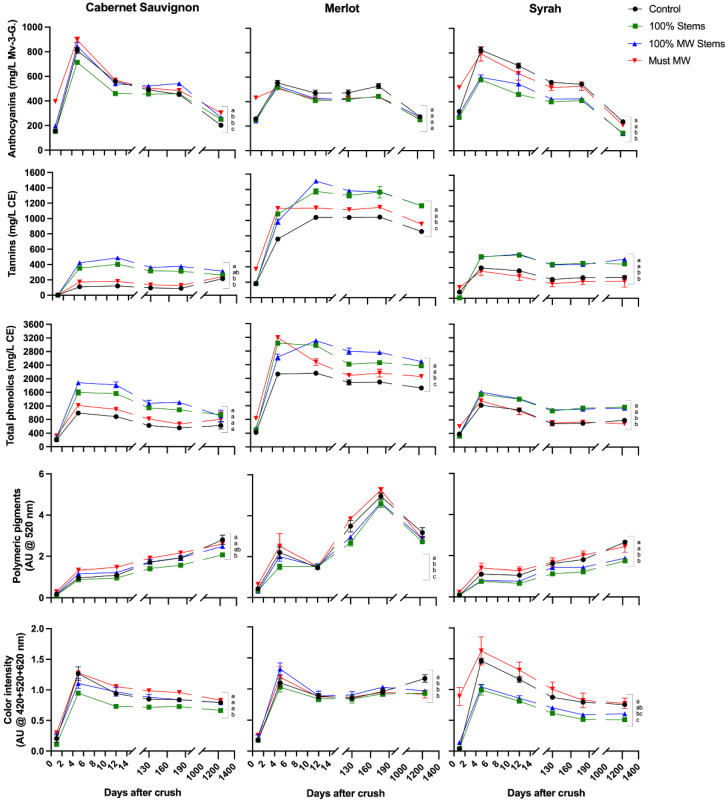
Evolution during winemaking, early and extended bottle aging of selected phenolic classes and wine color in the wines of the 2017 vintage. Different letters in the last sampling point (12 months of bottle aging), indicate significant differences for Fisher’s LSD test and *p* < 0.05. Mv-3-G.: malvidin-3-glucoside equivalents; AU: absorbance units; CE: catechin-equivalents.

**Figure 4:**
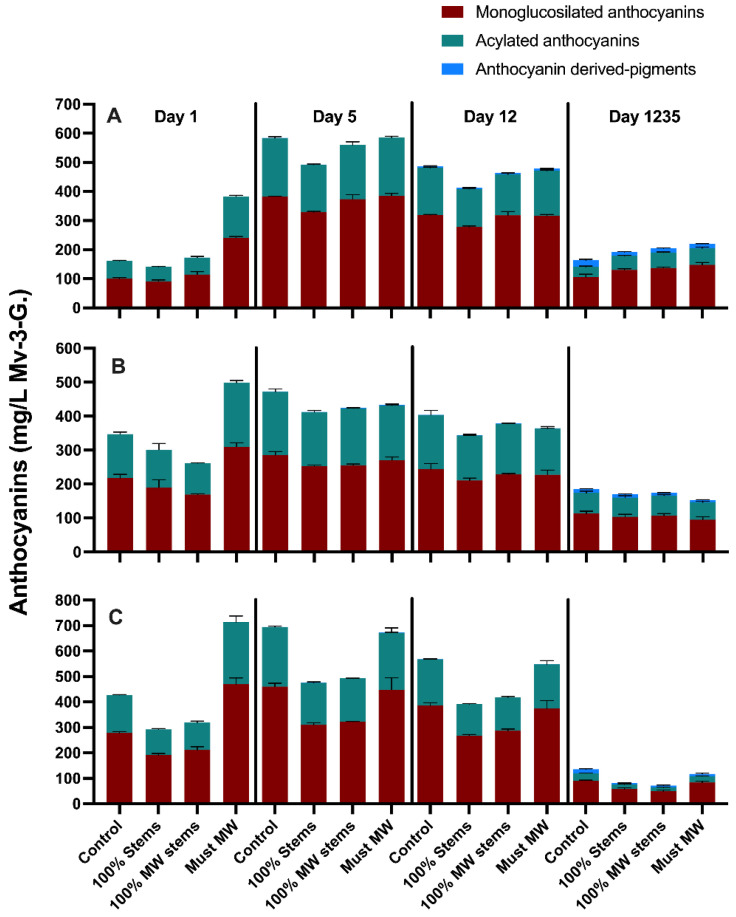
Evolution during winemaking and extended bottle aging (36 months) of monoglucosilated, acylated and anthocyanin-derived pigments. (**A**) Cabernet Sauvignon; (**B**) Merlot; (**C**) Syrah. Mv-3-G.: malvidin-3-glucoside equivalents.

**Figure 5:**
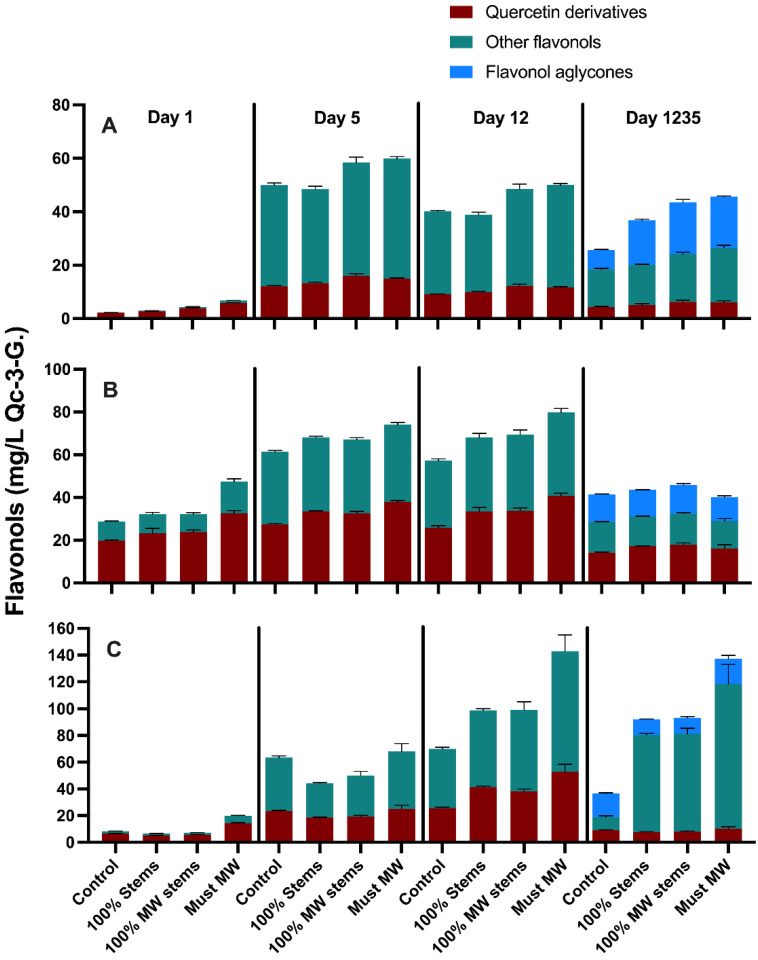
Evolution during winemaking and extended bottle aging (36 months) of quercetin derivates, other flavonols and flavonol aglycones. (**A**) Cabernet Sauvignon; (**B**) Merlot; (**C**) Syrah. Qc-3-G.: quercetin-3-glucoside equivalents.

The wines of the 2017 vintage were followed closely during winemaking, with the aim of assessing the extraction and evolution of the main phenolic classes throughout maceration and key winemaking stages up to 1230 days post-crushing (equating to 36 months of bottle aging) (Figure 3). Supplemental Table S2 shows the separate effects of the MW-based techniques on each cv., as well as the aggregate effect of each technique on all the three cvs. considered together. Finally, the detailed anthocyanin and flavonol composition of the wines, determined by HPLC-DAD-MS was also followed during the extraction (i.e., maceration) period (i.e., days 1, 5 and 12 post-crushing), and reassessed at the end of bottle aging (36 months) (Figure 4 and Figure 5, respectively). 

Expected trends in the extraction patterns of anthocyanins, tannins and polymeric pigment formation throughout winemaking and bottle aging in the 2017 wines were in accordance with current models of phenolic extraction into wine [31,32,33]. For example, anthocyanins peaked at day 6 post-crushing, a trend that was followed by a relatively sharp decrease thereafter. Tannin extraction progressed more slowly, and, concomitantly, polymeric pigment formation occurred progressively throughout winemaking and bottle aging, especially in the case of Cabernet Sauvignon and Syrah wines (Figure 3). These trends were also reflected in the evolution of monomeric anthocyanins and in the progressive formation of anthocyanin-derived pigments (Figure 4 and Supplemental Table S4).

In Cabernet Sauvignon wines, anthocyanins were initially higher in Must MW wines, and remained comparatively higher during winemaking and aging. Previous research with Cabernet Sauvignon wines has shown that application of MW led to a 40% decrease in the activity of polyphenol-oxidases [13], which would help preserve anthocyanins against enzymatic oxidations. This, coupled with enhanced diffusion mediated by the high temperature generated by the MW process [11], may have resulted in enhanced anthocyanin extraction in Must MW wines. Analysis of the detailed anthocyanin composition of the wines confirmed this trend, whereby at day 1235 Must MW wines had significantly higher content of all anthocyanin classes (Figure 4A and Supplemental Table S4). 

Whereas anthocyanin evolution in Merlot wines was ostensibly unaffected by the winemaking treatments, in Syrah wines, the addition of stems (100% Stems and 100% MW Stems treatments) negatively affected anthocyanin extraction. This outcome runs counter to what was observed in Cabernet Sauvignon wines where stems were added in the 2016 vintage, possibly suggesting that the adsorptive capacity of Syrah stems on monomeric anthocyanins outweighed possible benefits related to tannin contribution from the stems. After 36 months of bottle aging, both Control and Must MW wines showed higher anthocyanin content than the remaining treatments. The detailed anthocyanin composition of the 2017 Syrah wines also reflected this trend whereby levels of monoglucosylated and acylated anthocyanins, as well as that of anthocyanin-derived pigments were higher in both Control and Must MW wines (Figure 4C). Overall, Must MW increased anthocyanins and anthocyanin-derived pigments albeit less so relative to Control wines (Supplemental Table S4).

Tannins were significantly higher in Merlot wines, with levels ranging from 900 to 1200 mg/L, well above those typically observed in the wines of this cv., which averages 559 mg/L CE (*n* = 197) [34]. Notwithstanding relative differences ascribed to each cv., tannin extraction and total phenolic content was significantly enhanced in 100% Stems and 100% MW Stems wines. In Merlot wines, tannins increased by 39% in both 100% Stems and 100% MW Stems wines relative to Control wines. In Syrah wines, these same treatments improved tannin content by 63% and 85%, respectively, relative to Control wines. On the other hand, Must MW wines did not show any improvement in tannin extraction for Cabernet Sauvignon and Syrah wines (Supplemental Table S2).

Extraction and evolution of total phenolics mirrored the discussed trends in tannin extraction discussed above. The determination of the detailed flavonol composition of these wines throughout winemaking and aging was of interest as flavonols act as copigmentation factors. Visually positive hyperchromic and bathochromic shifts in the visible spectrum of the resulting wines have been ascribed to copigmentation reactions, and these shifts signal more stable color during bottle aging [35]. Flavonols may also play a role in specific and potentially desirable mouthfeel properties, such as the perception of a velvety astringency subquality [36] and taste sensations such as bitterness [37]. Flavonols were generally highest in Syrah wines, intermediate in Merlot wines and the lowest in Cabernet Sauvignon wines (Figure 5 and Supplemental Table S5). The production of flavonol aglycones, which occurs progressively during winemaking due to acid hydrolysis of the glycosidic moiety in the flavonol structure [38], was also clearly observed by the end of the study at day 1235 post-crushing (Figure 5). In general, the three winemaking techniques resulted in noticeable increases in wine flavonol content, especially in the 100% MW Stems and Must MW treatments. Must MW improved flavonol content by 278% at day 1235 post-crushing in Syrah wines (Figure 5C). Moreover, 100% MW Stems treatment improved flavonol content by 72% in Cabernet Sauvignon wines (Figure 5A). A previous study of three Pinot noir clones using stems treated with MW prior to alcoholic fermentation reported no effect of stem additions on flavonol content [20]. Together, these results suggest that stems of different cvs. may have qualitative and quantitative differences in their extractable phenolic pool [39], flavonol among them. When the results of all the three cvs. were pooled together, the most effective treatment for flavonol extraction was the Must MW treatment, yielding an overall 60% enhancement of the total flavonol content relative to Control wines (Supplemental Table S5). The MW treatment may have favored the diffusion and mass-transfer process of flavonols into the wines through the generation of heat. In the case of MW Stems treatment, heat-driven extraction of stem-derived flavonols into the wines may have increased wine flavonol content. The latter is most likely the case, as stems have been reported to contain flavonols, especially quercetin and kaempferol derivatives [17]. 

Polymeric pigments increased progressively throughout aging, although Merlot wines had a dip in their content towards the end of the study (Figure 3). Generally, 100% Stems and 100% MW Stems treatments resulted in wines with lower polymeric pigment contents, suggesting the added stems did not affect the evolution of these compounds. Whereas the Must MW treatment wines were higher in polymeric pigment than the stem-added wines, they only had comparable polymeric pigment content to Control wines. Thus, none of the winemaking techniques herein applied was able to favor polymeric pigment formation in the 2017 wines (Supplemental Table S2).

**Figure 6:**
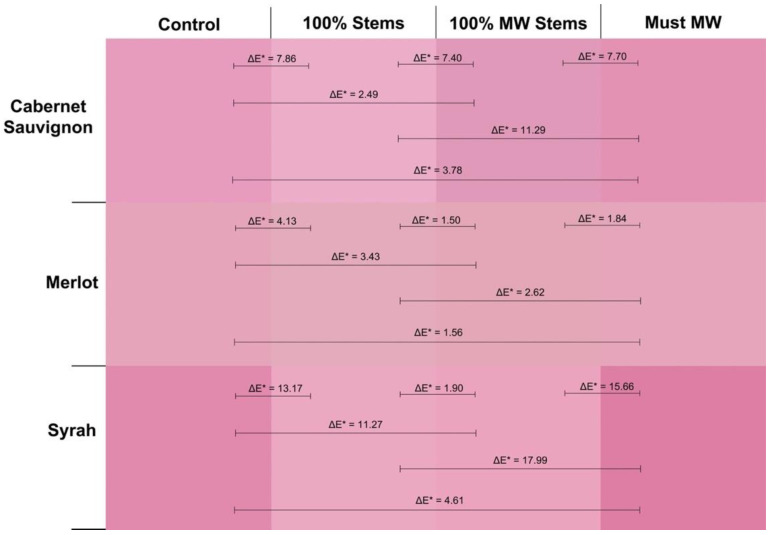
Visual depiction of the actual color of the 2017 wines of the three cvs. and four winemaking treatments as seen through a 1 mm path length quartz cuvette at the time of pressing (day 12). ΔE* values are shown between each pair of treatments for a given cv.

Wine color intensity, which results from the summation of the three discrete absorbances of 420, 520 and 620 nm was generally higher in Control and Must MW wines, and generally the lowest in the stem-added wines (Figure 3). Thus, the color of stem-added wines was again negatively affected by stem additions irrespective of the cv. The color panels shown in Figure 6 confirmed that Control and Must MW wines exhibited noticeable and improved chromatic characteristics relative to the stem-added wines at pressing. For Merlot wines color differences between Control and Must MW wines were small and based upon ΔE* values that are indistinguishable by the human eye (Figure 6, middle panel), in Cabernet Sauvignon and Syrah wines ΔE* values of 3.78 and 4.61 CIELab units, respectively, indicate perceivable differences in favor of Must MW wines (Figure 6, top and bottom panels, respectively). As Must MW wines for Cabernet Sauvignon and Syrah did not result in enhanced tannin extraction, but did improve flavonol retention, it is possible that these early positive effects on color saturation in Must MW wines are due to enhanced copigmentation, which in turn resulted in an hyperchromic shift in the resulting wines. It is unclear why this putative copigmentation effect was not observed in MW Stem wines, which were also higher in flavonols. Nonetheless, flavonols represent the most efficient copigments for anthocyanins, resulting in bathochromic (i.e., purple hues), and hyperchromic shifts (i.e., higher saturation), as shown elsewhere [40]. These enhancements can be confirmed and observed in Figure 6. 

#### 2.3.3. 2018 Vintage Wines

During the 2018 vintage, the application of the Must MW treatment was discontinued, and instead stem additions at two selected rate additions (50 and 100%) and processed with and without prior treatment with MW, were pursued. The decision to study the effect of stems at the 50 and 100% rate additions was based on previously observed chemical effects in wines made with stem additions, particularly those pertaining to enhanced tannin extraction (and, potentially, positive mouthfeel characteristics), in a more or less proportional fashion to the amount of stems added to the wines [19,20,41]. 

**Figure 7:**
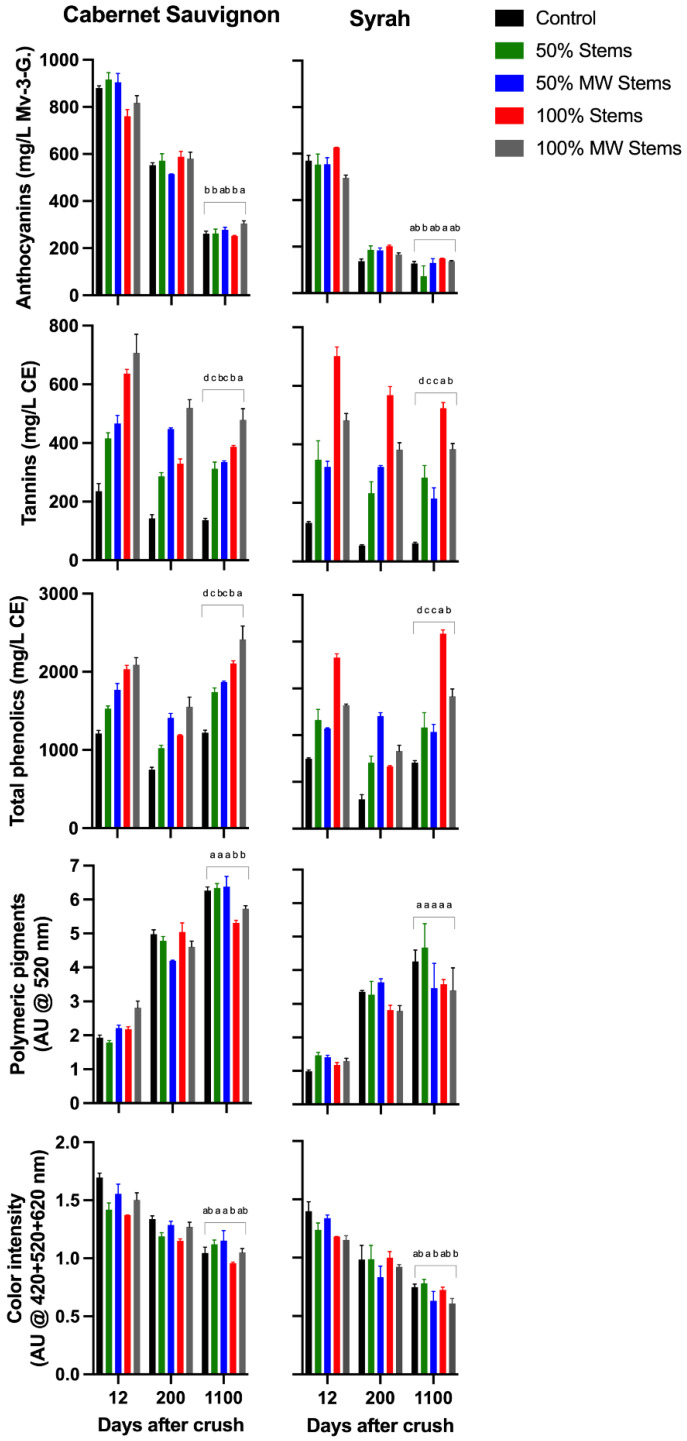
Evolution during winemaking and extended bottle aging of selected phenolic classes and wine color in the wines of the 2018vintage. Different letters in the last sampling point (12 months of bottle aging), indicate significant differences for Fisher’s LSD test and *p* < 0.05. Mv-3-G.: malvidin-3-glucoside equivalents; AU: absorbance units; CE: catechin-equivalents.

Figure 7 shows the evolution of the major phenolic classes at key times during the red winemaking process, namely at pressing (day 12), after completion of MLF (day 200 post-crushing), and after an extended period of bottle aging (day 1100 post-crushing, equating to 32 months of bottle aging). Supplemental Table S3 presents the separate effect of each cv. (Cabernet Sauvignon and Syrah), and those of the winemaking techniques, as well as the aggregate effect of the winemaking techniques in the two cvs. under study in 2018. 

Whereas the overall tannin content of the wines was generally comparable between the Cabernet Sauvignon and Syrah wines, anthocyanins were generally higher in Cabernet Sauvignon wines. As expected, anthocyanins decreased progressively after pressing, notably after completion of MLF. The application of the 100% MW Stems treatment significantly enhanced anthocyanins in Cabernet Sauvignon, but this treatment had no effect on anthocyanins in Syrah wines.

As observed in the two previous vintages, the most dramatic effects of the stem-based winemaking techniques were seen in the tannin content of the wines. At the last sampling point (day 1100 post-crushing), the 100% MW Stems treatment resulted in Cabernet Sauvignon wines with a 250% enhancement and the 100% Stems treatment resulted in Syrah wines with 743% enhancement of the tannin content relative to Control wines (Figure 7 and Supplemental Table S3). The latter represents an 8-fold increase in the tannin content of these Syrah wines that received 100% stems. The 50% stem-added treatments generally produced intermediate increases relative to Control wines, which were, nonetheless, highly significant (Supplemental Table S3). 

Whereas total phenolics generally mirrored the discussed results for tannins, and remained relatively steady throughout winemaking, polymeric pigments increased progressively in all the wines. As observed in the previous two vintages, polymeric pigment formation was generally and negatively affected by stem additions at a 100% rate in Cabernet Sauvignon wines, whereas in Syrah wines there was no effect of any winemaking technique on polymeric pigment formation. Likewise, none of the winemaking techniques succeeded at enhancing wine color relative to that obtained in Control wines in 2018 (Figure 7 and Supplemental Table S3). 

**Figure 8:**
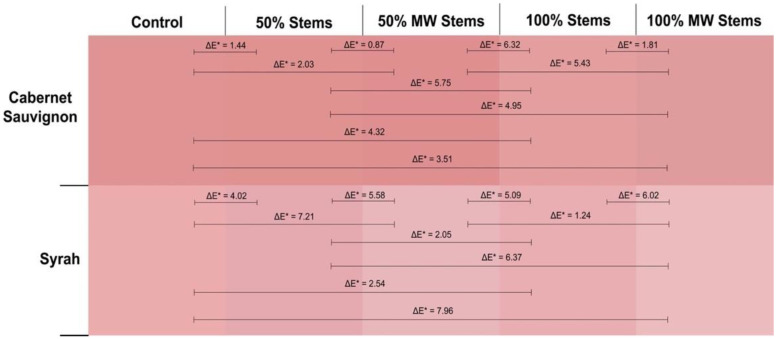
Visual depiction of the actual color of the 2018 wines of the three cvs. and four winemaking treatments as seen through a 1 mm path length quartz cuvette at day 1100 (32 months of bottle aging). ΔE* values are shown between each pair of treatments for a given cv.

These trends were also confirmed by the color panels of the wines that were generated at the end of the study (Figure 8). In these panels, Cabernet Sauvignon Control wines were visually more saturated than 100% Stems and 100% MW Stems wines, with ΔE* values of 4.32 and 3.51 CIELab units, respectively, in favor of Control wines (Figure 8, top panel). For Syrah wines, this trend was amplified, as the comparison of the saturation of Control wines against that of 100% MW Stems wines resulted in a ΔE* value of 7.96 CIELab units, clearly indicating much lower color saturation of the 100% MW Stems Syrah wines at day 1100 post-crushing.

### 2.4. Sensory Analysis of the 2016 Vintage Wines

**Figure 9:**
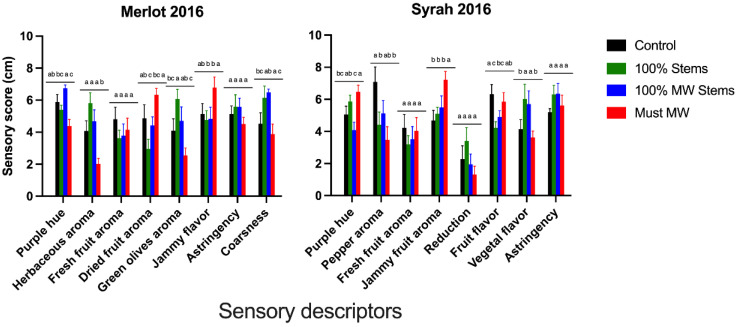
Sensory scores (10 cm unstructured line scale) of Merlot and Syrah wines from the 2016 vintage assessed by a sensory panel. Different letters within a sensory descriptor indicate significant differences for Fisher’s LSD test and *p* < 0.05.

Figure 9 shows the result of a sensory analysis carried out in two of the 2016 wines, Merlot and Syrah, by a panel of experienced tasters that were part of the winemaking technical team of a large winery in California. Eight different descriptors were selected by consensus prior to rating the wines using a 10 cm unstructured line scale.

In the 2016 Merlot wines, only 100% MW Stems wines showed increased purple hue, though this increase was not significant relative to Control wines (Figure 9). The color panels shown in Figure 2 confirm, however, the overall higher color saturation in 100% MW Stems wines. With regards to aroma attributes for Merlot wines, which included herbaceous, fresh fruit, dried fruit and green olives aroma, none of the treatments produced noticeable aromatic differences, with the exception of the Must MW treatment, for which wines the sensory panel reported a decrease perception of herbaceous aroma. From this sole perspective, the application of Must MW could be considered as a tool to curb the predominance of herbaceous and/or vegetal aromas in Merlot. Notably, neither the addition of green stems nor the addition of MW stems increased the perception of herbaceous aromas, as it would have been otherwise expected. Must MW wines, on the other hand, were perceived as having enhanced jammy flavor (relative to the stem-added wines), which could be the result of the heat applied during the MW process to the musts. Moreover, this treatment significantly reduced the perception of coarseness. Coarseness is a tactile sensation defined as a rough texture on the palate, and by analogy, related to the feel of coarser grade emery paper. Perceived coarseness correlates positively with the proportion with the degree of galloylation of tannins and inversely with the proportion of trihydroxylated subunits in the tannin structure [42]. It is thus possible that the Must MW treatment may have resulted in the extraction of epigallocatechin subunits by favoring skin tannin extraction. Remarkably, whereas large increases in protein precipitable tannins were observed in Merlot wines upon addition of stems (Figure 1 and Supplemental Table S1), these comparatively higher tannin levels did not directly translate into a higher perceived astringency in the resulting wines.

The Must MW treatment applied to Syrah resulted in enhanced purple hue in the finished wines, which was also confirmed in the color panels presented in Figure 2 (bottom panel). Regarding the aroma composition, which included pepper (considered a varietal aroma in Syrah), fresh fruit, jammy and reduction aromas, Control wines showed higher perceived pepper aroma. Thus, from this sole perspective, Control wines were the most representative of the varietal character of Syrah wines. Like what was found for jammy flavor in the case of Merlot wines, the Must MW treatment increased the perception of jammy fruit aroma in Syrah wines. In contrast with what was observed in Merlot wines, the treatments based upon stem addition increased the perception of vegetal flavor, relative to Control and Must MW Syrah wines. This enhanced perception of vegetal flavors can be the result of enhanced extraction and retention of pyrazines, which are important components of the vegetal character associated with stems [18]. However, it is unclear why these aromas did not prevail in Merlot wines treated with stems. Lastly, and despite similar trends of enhanced tannin extraction observed upon stem additions in Syrah wines, no effect on the perceived astringency on the finished wines was observed.

Overall, some practical insights can be gleaned from these sensory findings. First, that the widespread concern of extremely herbaceous and/or vegetal aromas being extracted from non-lignified (i.e., green) stems seem to be overmagnified, though the extent of the impact of these aromas is clearly contingent upon the cv. That is, there was a detectable vegetal/herbaceous aroma in Syrah wines treated with stems but there was no such effect in Merlot wines treated similarly. Second, that the application of Must MW can curb some of these aromas in cvs. such as Merlot, but they may be replaced by jammy and/or cooked fruit aromas. Third, although there were large increases in tannins associated with stem inclusions during maceration, these additions were necessarily associated with increased perception of coarseness or astringency of the resulting wines.

## 3. Materials and Methods

### 3.1. Grapes and Vineyard Sites

The present study spanned three consecutive but diverse vintages in the Central Coast of California (USA), namely 2016, 2017 and 2018 [43], and sourced Cabernet Sauvignon (clone 8), Merlot (clone 3), and Syrah (clone 877). Cabernet Sauvignon and Syrah were sourced from Lago and Alta Loma vineyards, respectively, in the Arroyo Seco AVA of Monterey County (Greenfield, CA, USA), whereas Merlot was sourced from the Sunnybrook Ranch, in the Paso Robles AVA of San Luis Obispo County (Paso Robles, CA, USA). In all three cases, vines were drip-irrigated and trained in a vertical shoot positioning (VSP) system with two catch-wires. Weather data were obtained from the California Irrigation Information Management System (CIMIS). Cumulative growing degree days (GDD) for seasonal (1 April to 31 October) documentation were calculated using a baseline temperature of 10 °C and the daily average temperature. Fruit was manually harvested in 0.5 tons bins, in amounts ranging from 1.01 to 1.48 tons per grape cultivar during each harvest and immediately transported to the Research Winery of the Wine and Viticulture Department (California Polytechnic State University, San Luis Obispo, CA, USA), with processing taking place the same day of fruit reception. A total of 9.05 tons of fruit were processed over the three vintages. In all instances, stems showed no signs of lignification and were green at harvest time. Forty clusters (*n* = 40) were randomly taken from each grape cv. prior to crushing and hand de-stemmed immediately to determine Brix, pH, titratable acidity, yeast assimilable nitrogen, potassium, and composition percentage of stems (on a fresh weight basis relative to the whole cluster weight). Brix was measured with a handheld refractometer (Vee Gee Scientific, Kirkland, WA, USA). Titratable acidity was measured by titrating a known quantity of juice (5 mL) in a deionized water solution against 0.067 N NaOH (Fisher Scientific, Waltham, MA, USA) to a pH endpoint of 8.2 in accordance with an established procedure [44]. Yeast assimilable nitrogen (YAN) and L-malic acid were measured enzymatically from juice utilizing an analyzer (Y15 Automatic Analyzer, Admeo, Angwin, CA, USA), and commercially available kits (Biosystems, Barcelona, Spain).

### 3.2. Winemaking and Experimental Design

Upon arrival to the Research Winery, grapes were processed using a crusher-destemmer (Bucher Vaslin, Niederweningen, Switzerland), with the rollers of the crusher disengaged. The musts were placed separately in individual 60 L plastic fermenters (Speidel, Swabia, Germany), with each fermenter receiving 50 kg (± 0.1 kg) of must. Immediately after crushing, 50 mg/L of SO_2_ was added to each fermenter and incorporated with a gentle, 30 s punch-down. Diammonium phosphate (Fermaid K, Lallemand, Rexdale, ON, Canada), was added to raise the yeast assimilable nitrogen to 300 mg/L prior to alcoholic fermentation in all cases. Musts were inoculated with a commercial strain of *Saccharomyces cerevisiae* (EC-1118, Lallemand, Rexdale, ON, Canada), at a rate of 30 g/hL. In the 2016 wines, malolactic bacteria *Lactobacillus plantarum* (ML Prime; Lallemand, Quebec, Canada) was added one day after yeast inoculation at a rate of 10 g/hL. For the 2017 and 2018 wines, malolactic bacteria *Oenococcus oeni* (VP41, Lallemand, Quebec, Canada), was added one day after yeast inoculation at a rate of 20 g/hL.

The following treatments were established in the 2016 and 2017 vintages in triplicate fermentations (*n* = 3): Control wines were produced following a standard winemaking procedure consisting of a total maceration time of 12 days during which two, 2 min punch-downs per day (morning and afternoon), were applied. For the Must MW treatments (Must MW), musts were microwaved at 1200 Watts for 10 min (reaching a temperature at the core of the must of 40 °C), using a household microwave as previously described [20]. For the stem-added treatments, prior to addition to the fermenters or processing by MW, the stems were treated with ozonated water (Clearwater Tech, San Luis Obispo, CA, USA) at a dose of 5 ppm O_3_. Addition of 100% stems by weight (100% Stems) was achieved by placing clean stems at the bottom of the 60 L fermenters in quantities according to the percentage of stems originally present in the clusters at harvest (Table 1). Addition of 100% stems by weight after microwave treatment (100% MW Stems), was achieved by microwaving the stems at 1200 Watts for 5 min (~400 Watts/kg), reaching an average temperature of approximately 57 °C. The MW-treated stems were then placed at the bottom of the 60 L fermenters in quantities according to the percentage of stems originally present in the clusters at harvest (Table 1). As with Control wines, cap management was kept strictly constant and consisted of two, 2 min punch-downs per day (morning and afternoon) and maceration time was set to 12 days for the Must MW, 100% Stems and 100% MW Stem treatments.

In 2018, the following experimental conditions established in triplicate fermentations (*n* = 3): Control, 100% Stems, and 100% MW Stems wines were produced strictly following the same protocol established in the 2016 and 2017 vintages for Control, 100% Stems and 100% MW Stem wines, respectively. In addition, two new treatments, consisting of inclusions of 50% stems (50% Stems) and of 50% stems after MW (50% MW Stems), were included following the same procedures previously detailed for the MW treatments. As in the 2016 and 2017 vintages, maceration time was set to 12 days of maceration time, after which the free run wines were transferred to 20 L glass carboys fitted with airlocks. The end of malolactic fermentation (MLF) was confirmed (≤0.2 g/L malic acid, Table 2) by enzymatic determination of L-malic acid (Y15 Automatic Analyzer, Admeo, Angwin, CA, USA), and commercially available kits (Biosystems, Barcelona, Spain), after which the wines received an addition of 25 mg/L of SO_2_. Wines were cold stabilized at 5 °C for 45 days, racked off the lees and bitartrate crystals and subsequently adjusted to 0.3 mg/L molecular SO_2_. The wines of the 2016, 2017 and 2018 vintages were bottled in February 2017, 2018, and 2019, respectively, using DIAM 5 micro-agglomerated cork closure (G3 Enterprises, Modesto, CA, USA; oxygen transmission rate: 0.4 mg/bottle/year; oxygen initial release: 1.3 mg), stored in vertical position and kept in cellar-like conditions (~12 to 14 °C) until analysis.

### 3.3. Wine Basic Chemical Composition

Wine titratable acidity (TA) and pH were measured following the same method detailed above for determination of juice TA and pH. Ethanol (% *v*/*v*), was measured by near-infrared spectroscopy using a Alcolyzer Wine M/ME analysis system (Anton Paar, Graz, Austria). Acetic acid, glucose, fructose, malic acid, and lactic acid were determined enzymatically using commercial enzymatic analysis kits (Admeo, Biosystems Group, Hollister, CA, USA). Free and total SO_2_ concentration were determined by the aspiration method [44].

### 3.4. Wine Spectrophotometric Analysis

Spectrophotometric measurements included analysis of phenolic compounds and color parameters and were performed to evaluate the effect of the winemaking techniques applied herein on the evolution of phenolic compounds and chromatic characteristics during selected winemaking and bottle aging stages. In the 2016 vintage the wines were analyzed after 3 months and 12 months of bottle aging, whereas CIELab coordinates were determined at pressing. For the 2017 vintage, the wines were analyzed throughout maceration, bottle aging, and up to 1230 days post-crushing (equivalent to 36 months of bottle aging), whereas CIELab coordinates were determined at pressing. In the 2018 vintage, wines were analyzed at pressing, after completion of MLF and up to 1100 days post-crushing (equating to 32 months of bottle aging), whereas CIELab coordinates were determined at the end of the bottle aging period (day 1100). In all cases, wine samples were centrifuged at 15,000 g in a microfuge (model 5415D; Eppendorf, Hamburg, Germany), and the supernatant transferred into clean 1 mL Eppendorf tubes prior to analysis. Anthocyanins and total polymeric pigments [herein defined as the sum of small polymeric pigments (SPP) and large polymeric pigments (LPP)], were measured as previously reported [45]. Tannins were analyzed by protein precipitation [46]. Wine color intensity was determined by placing an aliquot of undiluted wine samples in 1 mm path length quartz cuvettes, and the absorbances at 420, 520 and 620 nm were recorded. Wine color intensity was calculated as the sum of absorbances at 420, 520 and 620 nm, as previously detailed [47]. CIELab color coordinates were determined in 1 mm path length quartz cuvettes. CIELab coordinates were calculated using the Cary WinUV color software (version 6.0, Startek Technology, Boronia, Vic., Australia) under a D65 illuminant [48]. To explore overall chromatic differences between treatments, the CIELab color difference (ΔE*) between a given pair of wines was calculated as the Euclidean distance between two points (r and s), in the three-dimensional CIELab space using the following equation: ΔE*r,s = [(ΔL*r,s)2 + (Δa*r,s)2 + (Δb*r,s)2]1/2 as previously described [49]. Spectrophotometric measurements were made with a Cary 60 UV-Vis spectrophotometer equipped with a 18-sample cell auto-sampler (Agilent Technologies, Santa Clara, CA, USA).

Visual depiction of the wines at pressing for the 2016 and 2017 vintage wines, and after extended bottle aging in 2018 wines was accomplished by imputing L*, a* and b* values into a color converter (www.nixsensor.com, accessed on 12 December 2021), considering the standard CIELab conditions (10° standard observer and the illuminant D65), and recorded in a 1 mm path length quartz cuvette.

### 3.5. Wine Analysis by HPLC-Diode Array Detector-MS

The wines of the 2017 vintage were analyzed by HPLC-diode array detector (DAD) with peak identity confirmed by MS throughout maceration (days 1, 5 and 12) and after 36 months of bottle aging. Prior to analysis, the wines were centrifuged for 10 min at 15,000 g (Eppendorf 5430 R, Hamburg, Germany) and filtered through a 0.45 μm membrane (Sartorius, Goettingen, Germany). The wines were analyzed in an Agilent 1100 series HPLC system coupled to a DAD (Agilent Technologies), as previously described [50], with minor modifications. Separation occurred in a Zorbax SB-C18 column (4.6 mm × 150 mm, 3.5 μm particle size; Agilent Technologies) thermostated at 40 °C and protected by a guard column of the same packing material. Peak identity was confirmed using a Waters Acquity I-Class ultra-performance liquid chromatography system connected to an AB Sciex 4000 Q-Trap MS/MS (Waters, Milford, MA, USA). The column eluent, under the same conditions described earlier, was directed to the mass spectrometer operating in positive ionization mode, and compounds were detected by multiple reaction monitoring. Monomeric anthocyanins were quantified using malvidin-3-glucoside chloride as standard (Extrasynthèse, Lyon, France), and a standard calibration curve (R^2^ = 0.99). Flavonols were quantified using quercetin-3-glucoside (Sigma-Aldrich, St Louis, MO, USA), as standard and a standard calibration curve (R^2^ = 0.99).

### 3.6. Sensory Analysis

The Merlot and Syrah wines of the 2016 harvest were analyzed during a single session after 3 months of bottle aging. The panel, composed by 8 winemakers (ages ranging from 25 to 49 years, 3 females), was part of a large winery and all of them had extensive experience in wine sensory analysis. Terminology agreement, definition and consensus were established before the evaluation session. Briefly, panelists defined by consensus 8 sensory attributes, including visual aspects (purple hue), orthonasal aroma attributes, retronasal aroma attributes (i.e., flavor), as well mouthfeel attributes (astringency and coarseness). During the evaluation session, the intensity of each attribute was assessed using a non-structured 10 cm line scale containing two reference points located at 1 cm of each end of the line. Wines and their replicates (*n* = 3) were presented in aliquots of 25 mL placed in ISO wine glasses covered with plastic lids to trap volatiles, following a full randomize serving order. To minimize sensory carry-over, panelists were asked to rinse their mouth with mineral water and eat a cracker between samples following a sip and spit protocol.

### 3.7. Statistical Analysis

Wines were produced in triplicate fermentations (*n* = 3) across all the three vintages under study. The fruit data of the 2016 and 2017 vintages were analyzed by a fixed-effect one-way analysis of variance (ANOVA), whereas the 2018 fruit data were analyzed by a Student t-test (*p* < 0.05). The basic chemical, phenolic, color and sensory composition of the wines was analyzed by a one-way ANOVA. For the basic chemical, phenolic and color composition, these were further reassessed by a two-way ANOVA separating the effect of cv. and winemaking treatment, as well as their respective interaction. The HPLC-DAD-MS data were analyzed by a one-way ANOVA and reassessed by a three-way ANOVA separating the effect of cv., winemaking treatment, and time (days after crush), as well as their respective interactions. In all cases, Fisher’s LSD test was used as a post hoc comparison of means with a 5% level for rejection of the null hypothesis. Data were analyzed with XLSTAT (Addinsoft, Paris, France), and all graphical representations were prepared with GraphPad Prism software version 9.0 (GraphPad Soft-ware, San Diego, CA, USA).

## 4. Conclusions

MW technology was applied to Cabernet Sauvignon, Merlot and Syrah grapes from the Central Coast of California during two relatively cool vintages (2016 and 2018), and a very warm vintage (2017). Stems were not lignified at harvest time and were added after MW treatment. Musts were also treated with MW prior to alcoholic fermentation.

The most noteworthy chemical effects associated with stem additions were large increases in the tannin content of the wines (up to 8-fold for Syrah wines in 2018), coupled with losses of color and anthocyanins, especially when green stems were added to Syrah wines. The application of MW to the stems prior to alcoholic fermentation somewhat curbed such losses, though it did not consistently improve color relative to Control wines. The most notable effect of the Must MW treatment was to increase flavonols, which could bear positive sensory implications for wine’s color, mouthfeel, and bitterness.

Generally, none of the techniques herein applied positively affected color evolution and polymeric pigment formation. Therefore, if color is a stylistic concern for a given wine or cv., it should be kept in mind that stem additions may negatively affect color. Special consideration should be given to cvs. with inherently low levels of anthocyanins (such as Nebbiolo or Pinot noir), in which stem additions will further reduce wine color saturation.

Less pronounced effects on stem-derived tannin extraction were observed in the comparatively warmer 2017 vintage. During warm to hot vintages, stem inclusions can be used as a stylistic tool to boost tannins and decrease ethanol content, potentially enhancing perceived balance and freshness in the resulting wines. Conversely, in cool vintages that can afford a long ripening period, and possibly more hang time for the accumulation of stem-derived tannins, stems should be used cautiously as they may result in large and possibly unintended increases in the tannin content of the wines.

The sensory analysis of the Merlot and Syrah wines of the 2016 vintage revealed that although the tannin content of stem-added wines was substantially higher, it did not result in enhanced perception of astringency or coarseness. Stem additions did not increase perceived herbaceous aromas in Cabernet Sauvignon and Merlot wines either, but they did in Syrah wines. Overall, stem additions prior to alcoholic fermentation had more detrimental effects on Syrah than on Cabernet Sauvignon and Merlot wines, resulting in lower color saturation and enhanced (and possibly excessive) tannin extraction, and perception of herbaceous aromas. The Must MW treatment decreased the perception of coarse mouthfeel and herbaceous aromas but increased jammy aromas and flavors in the resulting wines. Thus, the Must MW may be considered as a tool to lessen herbaceous and vegetal aromas and coarseness mouthfeel perception in cooler vintages.

When considering the application of MW technology to stems or musts prior to fermentation, careful thought should be given to the style of wine that is sought to be produced. The application of MW needs to accent the typical focus of each cv., be appropriate for the vintage and optimize the interval between winemaking and released of the wine. Moreover, blending of wines from fully treated and untreated portions may diminished the tradeoff in sensory qualities observed herein and, in turn, increase complexity.

## Figures and Tables

**Table 1:** Initial grape chemistry, harvest dates, growing degree days, and percentage of stems in Cabernet Sauvignon, Merlot and Syrah grapes at the time of harvest. Values represent the average of 3 (*n* = 3) field replicates.

Vintage	Growing Degree Days (°C)	Cultivar	Harvest Date	Brix	pH	Titratable Acidity (g/L Tartaric Acid)	L-malic Acid (g/L)	Yeast Assimilable Nitrogen (mg/L as N)	Potassium (mg/L)	Percentage of Stems
2016	1557	Cabernet Sauvignon	29-Oct-2016	24.6 b ^(^*^)^	3.56 b	6.52 a	2.38 a	176 a	2410 ab	6.51 a
	1594	Merlot	27-Oct-2016	23.0 c	3.86 a	4.42 b	1.42 b	115 a	2670 a	3.79 c
	1557	Syrah	29-Sep-2016	25.0 a	3.56 b	6.81 a	2.71 a	196 a	2030 b	4.46 b
*p*-value				**<0.0001**	**0.0061**	**0.0021**	**0.0015**	0.1250	**0.0010**	**<0.0001**
2017	1734	Cabernet Sauvignon	24-Oct-2017	23.5 b	3.48 c	6.11 a	2.44 a	197 a	1940 b	5.72 a
	1803	Merlot	9-Nov-2017	24.2 ab	4.06 a	4.12 b	1.69 b	75 b	3060 a	4.16 b
	1734	Syrah	4-Oct-2017	25.3 a	3.75 b	4.71 a	2.38 a	145 ab	2090 a	5.64 a
*p*-value				**0.0010**	**<0.0001**	**0.0035**	**0.0010**	**0.0050**	**0.0015**	**<0.0001**
2018	1558	Cabernet Sauvignon	9-Nov-2018	25.4 a	3.39 b	6.62 a	2.45 b	196 a	1680 a	6.01 a
	1558	Syrah	15-Oct-2018	26.3 a	3.60 a	5.52 b	2.80 a	88 b	1700 a	5.12 b
*p*-value				0.6183	**<0.0001**	**<0.0001**	**<0.0001**	**<0.0001**	0.1648	**<0.0001**

^(^*^)^ For the 2016 and 2017 vintages, different letters within a column and a same vintage indicate significant differences for Fisher’s LSD test and *p* < 0.05. For the 2018 vintage, different letters within a column indicate significant differences for the Student t-test and *p* < 0.05. Significant *p*-values are shown in bold fonts.

**Table 2:** One-way and two-way analyses of variance (ANOVA) separating the effects of cultivar and winemaking treatment on the basic chemical composition of Cabernet Sauvignon, Merlot and Syrah wines from the 2016 vintage. Values represent the mean of three tank replicates (*n* = 3).

One-way ANOVA								
Cultivar	Winemaking Treatment	Ethanol (% *v*/*v*)	pH	Titratable Acidity (g/L Tartaric Acid)	Glucose + Fructose (g/L)	Acetic Acid (g/L)	Malic Acid (g/L)	Lactic Acid (g/L)
Cabernet Sauvignon	Control	15.52 a ^(^*^)^	3.93 b	5.64 a	0.31 b	0.33 a	0.15 a	1.45 b
	100% Stems	14.55 c	4.01 ab	5.39 b	0.04 d	0.33 a	0.13 a	1.72 a
	100% MW Stems	14.76 b	4.05 a	5.46 b	0.20 c	0.33 a	0.12 a	1.66 a
	Must MW	15.61 a	3.98 ab	5.48 b	0.34 a	0.41 a	0.14 a	1.29 c
	*p*-value	**<0.0001**	0.0702	**0.0215**	**<0.0001**	0.5713	0.5422	**<0.0001**
Merlot	Control	12.41 b	3.83 c	3.77 a	0.17 a	0.28 a	0.15 a	1.28 a
	100% Stems	12.17 c	3.88 bc	3.53 b	0.14 a	0.28 a	0.14 a	1.26 a
	100% MW Stems	12.34 bc	3.95 a	3.52 b	0.16 a	0.30 a	0.12 a	1.23 a
	Must MW	12.84 a	3.92 ab	3.78 a	0.17 a	0.39 a	0.12 a	0.95 b
	*p*-value	**<0.0001**	**0.0041**	**<0.0001**	0.3700	0.3285	0.6697	**<0.0001**
Syrah	Control	14.99 b	3.84 b	5.94 a	0.30 ab	0.30 a	0.07 b	1.68 a
	100% Stems	14.69 c	3.97 a	5.41 b	0.25 b	0.28 a	0.08 b	1.98 a
	100% MW Stems	14.66 c	3.98 a	5.38 b	0.24 b	0.27 a	0.08 b	2.04 a
	Must MW	15.42 a	3.88 b	5.90 a	0.39 a	0.34 a	0.14 a	1.61 a
	*p*-value	**<0.0001**	**0.0022**	**0.0002**	**0.0320**	0.6986	**0.0221**	0.1895
**Two-way ANOVA**								
Winemaking treatment	Control	14.30 b	3.86 c	5.12 a	0.25 b	0.30 ab	0.12 a	1.47 b
	100% Stems	13.80 d	3.95 b	4.77 b	0.14 d	0.29 b	0.11 a	1.65 a
	100% MW Stems	13.92 c	3.99 a	4.79 b	0.20 c	0.30 b	0.11 a	1.64 a
	Must MW	14.62 a	3.93 b	5.05 a	0.30 a	0.38 a	0.13 a	1.28 c
	*p*-value	**<0.0001**	**<0.0001**	**<0.0001**	**<0.0001**	0.5712	**0.0202**	**<0.0001**
**Main Effects and Interactions**								
Cultivar	*p*-value	**<0.0001**	**<0.0001**	**<0.0001**	**<0.0001**	0.6989	**0.0098**	**<0.0001**
Cultivar × Winemaking	*p*-value	**<0.0001**	**<0.0001**	**<0.0001**	**<0.0001**	0.5700	**0.0202**	**<0.0001**

^(^*^)^ Different letters within a column for each variety indicate significant differences for Fisher’s LSD test and *p* < 0.05. Significant *p*-values are shown in bold fonts.

**Table 3:** One-way and two-way analysis of variance (ANOVA) separating the effects of cultivar and winemaking treatment on the basic chemical composition of Cabernet Sauvignon, Merlot and Syrah wines from the 2017 vintage. Values represent the mean of three tank replicates (*n* = 3).

One-way ANOVA								
Cultivar	Winemaking Treatment	Ethanol (% *v*/*v*)	pH	Titratable Acidity (g/L Tartaric Acid)	Glucose + Fructose (g/L)	Acetic Acid (g/L)	Malic Acid (g/L)	Lactic Acid (g/L)
Cabernet Sauvignon	Control	13.03 b ^(^*^)^	3.80 d	5.35 ab	0.05 b	0.34 b	0.11 a	1.19 b
	100% Stems	12.42 c	3.95 b	5.01 b	0.15 a	0.39 a	0.12 a	1.29 a
	100% MW Stems	12.90 b	4.02 a	4.90 b	0.16 a	0.42 a	0.10 a	1.26 ab
	Must MW	13.56 a	3.91 c	5.60 a	0.17 a	0.43 a	0.11 a	1.25 ab
	*p*-value	**<0.0001**	**<0.0001**	**0.0256**	**<0.0001**	**0.0092**	0.2740	0.1185
Merlot	Control	15.45 a	4.03 b	5.35 a	0.32 a	0.28 c	0.11 a	1.26 b
	100% Stems	14.60 c	4.14 a	5.40 a	0.20 b	0.40 ab	0.09 ab	1.41 a
	100% MW Stems	15.22 b	4.14 a	5.20 a	0.33 a	0.44 a	0.08 bc	1.47 a
	Must MW	15.15 b	4.05 b	5.35 a	0.23 b	0.33 bc	0.07 c	1.29 b
	*p*-value	**<0.0001**	**<0.0001**	0.6926	**0.0004**	**0.0223**	**0.0091**	**<0.0001**
Syrah	Control	14.93 a	3.87 a	5.11 ab	0.23 a	0.29 c	0.16 a	1.40 b
	100% Stems	12.95 b	3.85 a	5.05 b	0.16 c	0.37 ab	0.12 b	1.59 a
	100% MW Stems	13.05 b	3.88 a	5.15 ab	0.17 c	0.35 b	0.12 b	1.59 a
	Must MW	14.98 a	3.89 a	5.40 a	0.20 b	0.40 a	0.14 ab	1.42 b
	*p*-value	**0.0011**	0.4813	0.1114	**<0.0001**	**0.0001**	**0.0120**	**0.0001**
**Two-way ANOVA**								
Winemaking treatment	Control	14.47 a	3.90 d	5.26 ab	0.20 b	0.30 b	0.12 a	1.28 c
	100% Stems	13.32 c	3.98 b	5.15 b	0.17 c	0.39 a	0.11 b	1.43 a
	100% MW Stems	13.72 b	4.01 a	5.08 b	0.22 a	0.40 a	0.10 c	1.44 a
	Must MW	14.56 a	3.95 c	5.45 a	0.20 b	0.38 a	0.11 bc	1.32 b
	*p*-value	**<0.0001**	**<0.0001**	**0.0146**	**<0.0001**	**<0.0001**	**<0.0001**	**<0.0001**
**Main effects and interactions**								
Cultivar	*p*-value	**<0.0001**	**<0.0001**	0.2940	**<0.0001**	**<0.0001**	**<0.0001**	**<0.0001**
Cultivar × Winemaking	*p*-value	**<0.0001**	**<0.0001**	**0.0146**	**<0.0001**	**<0.0001**	**<0.0001**	**<0.0001**

^(^*^)^ Different letters within a column for each variety indicate significant differences for Fisher’s LSD test and *p* < 0.05. Significant *p*-values are shown in bold fonts.

**Table 4:** One-way and two-way analysis of variance (ANOVA) separating the effects of cultivar and winemaking treatment on the basic chemical composition of Cabernet Sauvignon and Syrah wines from the 2017 vintage. Values represent the mean of three tank replicates (*n* = 3).

One-way ANOVA								
Cultivar	Winemaking Treatment	Ethanol (% *v*/*v*)	pH	Titratable Acidity (g/L Tartaric Acid)	Glucose + Fructose (g/L)	Acetic Acid (g/L)	Malic Acid (g/L)	Lactic Acid (g/L)
Cabernet Sauvignon	Control	14.88 a ^(^*^)^	3.77 d	5.50 a	0.39 a	0.23 b	0.03 ab	1.40 c
	50% Stems	14.60 b	3.79 cd	5.51 a	0.15 b	0.23 b	0.04 a	1.81 b
	50% MW Stems	14.57 b	3.82 c	5.41 ab	0.05 c	0.29 a	0.03 ab	1.98 ab
	100% Stems	14.25 c	3.89 b	5.14 b	0.03 c	0.31 a	0.02 b	2.05 a
	100% MW Stems	14.73 ab	3.98 a	5.25 ab	0.06 c	0.30 a	0.02 ab	2.06 a
	*p*-value	**0.0002**	**<0.0001**	0.1205	**<0.0001**	**<0.0001**	0.1381	**<0.0001**
Syrah	Control	13.69 a	3.57 c	6.34 a	0.05 a	0.42 c	0.01 a	2.18 a
	50% Stems	13.05 ab	3.63 b	6.11 a	0.03 a	0.54 a	0.01 a	1.87 b
	50% MW Stems	12.83 ab	3.67 a	5.65 b	0.03 a	0.45 bc	0.02 a	2.01 ab
	100% Stems	13.04 ab	3.69 a	6.14 a	0.05 a	0.50 ab	0.02 a	2.25 a
	100% MW Stems	12.23 b	3.68 a	5.73 b	0.03 a	0.51 ab	0.02 a	2.02 ab
	*p*-value	**0.0499**	**0.0007**	**0.0042**	0.5050	**0.0410**	0.8540	0.1100
**Two-way ANOVA**								
Winemaking treatment	Control	14.29 a	3.67 e	5.92 a	0.22 a	0.32 b	0.02 a	1.79 c
	50% Stems	13.82 b	3.71 d	5.80 ab	0.09 b	0.38 a	0.02 a	1.84 bc
	50% MW Stems	13.70 b	3.75 c	5.53 c	0.04 c	0.37 a	0.02 a	1.99 ab
	100% Stems	13.64 b	3.79 b	5.64 bc	0.04 c	0.40 a	0.02 a	2.15 a
	100% MW Stems	13.48 b	3.83 a	5.49 c	0.04 c	0.41 a	0.02 a	2.04 a
	*p*-value	**0.0090**	**<0.0001**	**0.0021**	**<0.0001**	**0.0020**	0.9100	**0.0020**
**Main effects and interactions**								
Cultivar	*p*-value	**<0.0001**	**<0.0001**	**<0.0001**	**<0.0001**	**<0.0001**	**0.0031**	**0.0011**
Cultivar × Winemaking	*p*-value	**0.0265**	**0.0003**	**0.0144**	**<0.0001**	**0.0082**	0.1681	**0.0011**

(*) Different letters within a column for each variety indicate significant differences for Fisher’s LSD test and *p* < 0.05. Significant *p*-values are shown in bold fonts.

## Data Availability

The data presented in this study are available in this article and in the supplemental files.

## Supplementary Materials

The following supporting information can be downloaded online: Supplemental Table S1: One-way and two-way analyses of variance (ANOVA) separating the effects of cultivar and winemaking treatment on the phenolic and color composition of Cabernet Sauvignon, Merlot and Syrah wines from the 2016 vintage after 12 months of bottle aging. Values represent the mean of three tank replicates (*n* = 3). Supplemental Table S2: One-way and two-way analyses of variance (ANOVA) separating the effects of cultivar and winemaking treatment on the phenolic and color composition of Cabernet Sauvignon, Merlot and Syrah wines from the 2017 vintage at day 1230 post-crushing (36 months of bottle aging). Values represent the mean of three tank replicates (*n* = 3). Supplemental Table S3: One-way and two-way analyses of variance (ANOVA) separating the effects of cultivar and winemaking treatment on the phenolic and color composition of Cabernet Sauvignon and Syrah wines from the 2018 vintage at day 1100 after crush (32 months of bottle aging). Values represent the mean of three tank replicates (*n* = 3). Supplemental Table S4: Three-way analysis of variance (ANOVA) separating the effects of cultivar, winemaking treatment, and time (days after crush), of the detailed anthocyanin composition (mg/L), of Cabernet Sauvignon, Merlot and Syrah wines from the 2017 vintage. Values represent the mean of three tank replicates (*n* = 3). Supplemental Table S5: Three-way analysis of variance (ANOVA) separating the effects of cultivar, winemaking treatment, and time (days after crush), of the detailed flavonol composition (mg/L), of Cabernet Sauvignon, Merlot and Syrah wines from the 2017 vintage. Values represent the mean of three tank replicates (*n* = 3).
