# Supplementary material for: Multi-Year Study of the Chemical and Sensory Effects of Microwave-Assisted Extraction of Musts and Stems in Cabernet Sauvignon, Merlot and Syrah Wines from the Central Coast of California
Source: Molecules. 2022 Feb 14;27(4):1270. doi: 10.3390/molecules27041270 (PMC8875832; doi:10.3390/molecules27041270)
Supplement: Supplementary file 1 [file molecules-27-01270-s001.zip › molecules-1570536 (-supplement ).pdf]

Multi-year study of the chemical and sensory effects of microwave-assisted extraction of musts and stems in Cabernet Sauvignon, Merlot and Syrah wines from the Central Coast of California

L. Federico Casassa [1,*], Paul A. Gannett[1], Nicholas Steele [1,2], and Robert Huff [1,3]

[1]Wine and Viticulture Department, California Polytechnic State University, San Luis Obispo, One Grand Ave., San Luis Obispo, CA 93407, USA.
[2]Jonata Winery, P.O. Box 191, Buellton, CA 93427, USA.
[3]Valkyrie Selections, LLC, Healdsburg, CA 95448, USA .

(*) Corresponding author: Dr. Federico Casassa (Email: lcasassa@calpoly.edu)

Supplementary Materials

Supplemental Table S1. One-way and two-way analysis of variance (ANOVA) separating the effects of cultivar and winemaking treatment on the phenolic and color composition of Cabernet Sauvignon, Merlot and Syrah wines from the 2016 vintage after 12 months of bottle aging. Values represent the mean of three tank replicates (n = 3).

Cultivar	Winemaking treatment	Anthocyanins (mg/L Mv-3-G.)	Tannins (mg/L CE)	Total phenolics (mg/L CE)	Polymeric pigments (AU @ 520 nm)	Color intensity (AU @ 420+520+620 nm)
**One-way ANOVA**						
Cabernet Sauvignon	Control	289.5 b(*)	258.4 d	1058.8 c	5.64 a	1.661 a
	100% Stems	239.4 b	523.2 b	1470.3 b	4.88 a	1.353 b
	100% MW Stems	352.5 a	619.3 a	1979.6 a	5.33 a	1.523 ab
	Must MW	374.1 a	339.0 c	1151.9 c	4.92 a	1.480 ab
	p-value	**0.0010**	**<0.0001**	**<0.0001**	0.2771	0.0968
Merlot	Control	56.1 ab	409.9 b	1151.7 c	2.98 a	0.832 a
	100% Stems	40.1 b	752.1 a	1676.9 ab	3.03 a	0.766 a
	100% MW Stems	68.0 a	838.2 a	1951.7 a	3.35 a	0.894 a
	Must MW	73.5 a	515.8 b	1425.4 bc	3.06 a	0.873 a
	p-value	0.0689	**0.0017**	**0.0268**	0.8133	0.7573
Syrah	Control	391.5 a	173.3 b	644.4 b	3.85 a	1.276 a
	100% Stems	364.4 a	430.2 a	1185.8 a	3.31 a	1.246 a
	100% MW Stems	316.5 a	427.7 a	1124.5 a	3.75 a	1.302 a
	Must MW	372.9 a	190.7 b	687.5 b	3.40 a	1.302 a
	p-value	0.2526	**<0.0001**	**<0.0001**	0.3124	0.8203
**Two-way ANOVA**						
Winemaking treatment	Control	245.7 a	280.5 d	951.6 c	4.15 a	1.257 a
	100% Stems	214.6 b	568.4 b	1444.3 b	3.74 a	1.122 b
	100% MW Stems	245.7 a	628.4 a	1685.3 a	4.14 a	1.240 ab
	Must MW	273.5 a	348.5 c	1088.3 c	3.79 a	1.219 ab
	p-value	**0.004**	**<0.0001**	**<0.0001**	0.1411	0.1306
**Main effects and interactions**						
Cultivar	*p*-value	**<0.0001**	**<0.0001**	**<0.0001**	**<0.0001**	**<0.0001**
Cultivar × Winemaking	*p*-value	**0.0013**	0.3106	0.1020	0.7776	0.5289

(*) Different letters within a column for each variety indicate significant differences for Fisher's LSD test and $p < 0.05$. Significant *p*-values are shown in bold fonts. Mv-3-G.: Malvidin-3-glucoside equivalents; AU: Absorbance units; CE: Catechin-equivalents.

Supplemental Table S2. One-way and two-way analysis of variance (ANOVA) separating the effects of cultivar and winemaking treatment on the phenolic and color composition of Cabernet Sauvignon, Merlot and Syrah wines from the 2017 vintage at day 1,230 post-crushing (36 months of bottle aging). Values represent the mean of three tank replicates (n = 3).

Cultivar	Winemaking treatment	Anthocyanins (mg/L Mv-3-G.)	Tannins (mg/L CE)	Total phenolics (mg/L CE)	Polymeric pigments (AU @ 520 nm)	Color intensity (AU @ 420+520+620 nm)
**One-way ANOVA**						
Cabernet Sauvignon	Control	202.3 c(*)	215.2 b	626.7 a	2.80 a	0.793 a
	100% Stems	251.1 b	262.0 ab	939.2 a	2.07 b	0.664 b
	100% MW Stems	265.5 b	313.4 a	907.9 a	2.48 ab	0.791 a
	Must MW	303.2 a	245.1 b	806.0 a	2.59 a	0.830 a
	p-value	**0.0004**	**0.0278**	0.4709	**0.0343**	**0.0427**
Merlot	Control	277.3 a	856.4 c	1720.6 c	3.17 a	1.170 a
	100% Stems	251.8 a	1190.4 a	2369.5 a	2.72 b	0.930 b
	100% MW Stems	273.9 a	1189.6 a	2493.8 a	2.86 ab	0.969 b
	Must MW	255.6 a	952.2 b	2055.8 b	2.87 ab	0.918 b
	p-value	0.3230	**<0.0001**	**<0.0001**	0.2040	**0.0156**
Syrah	Control	238.2 a	272.5 b	785.8 b	2.66 a	0.755 ab
	100% Stems	144.8 b	446.3 a	1170.6 a	1.74 b	0.512 c
	100% MW Stems	139.3 b	509.2 a	1141.8 a	1.87 b	0.605 bc
	Must MW	209.7 a	220.5 b	686.2 b	2.44 a	0.778 a
	p-value	**<0.0001**	**0.0025**	**<0.0001**	**0.0054**	**0.0170**
**Two-way ANOVA**						
Winemaking treatment	Control	239.3 ab	448.1 b	1044.4 b	2.88 a	0.906 a
	100% Stems	215.9 c	632.9 a	1493.1 a	2.18 c	0.702 c
	100% MW stems	226.2 bc	670.7 a	1514.5 a	2.41 bc	0.788 b
	MW must	256.2 a	472.6 b	1182.7 b	2.63 b	0.842 ab
	p-value	**0.001**	**<0.0001**	**<0.0001**	**<0.0001**	**<0.0001**
**Main effects and interactions**						
Cultivar	*p*-value	**<0.0001**	**<0.0001**	**<0.0001**	**<0.0001**	**<0.0001**
Cultivar × Winemaking	*p*-value	**<0.0001**	**<0.0001**	0.0679	0.2734	**0.0182**

(*) Different letters within a column for each variety indicate significant differences for Fisher's LSD test and $p < 0.05$. Significant *p*-values are shown in bold fonts. Mv-3-G.: Malvidin-3-glucoside equivalents; AU: Absorbance units; CE: Catechin-equivalents.

Supplemental Table S3. One-way and two-way analysis of variance (ANOVA) separating the effects of cultivar and winemaking treatment on the phenolic and color composition of Cabernet Sauvignon and Syrah wines from the 2018 vintage at day 1,100 after crush (32 months of bottle aging). Values represent the mean of three tank replicates (n = 3).

Cultivar	Winemaking treatment	Anthocyanins (mg/L Mv-3-G.)	Tannins (mg/L CE)	Total phenolics (mg/L CE)	Polymeric pigments (AU @ 520 nm)	Color intensity (AU @ 420+520+620 nm)
**One-way ANOVA**						
Cabernet Sauvignon	Control	261.5 b(*)	137.2 d	1222. d	6.26 a	1.045 ab
	50% Stems	262.8 b	312.2 c	1740.2 c	6.34 a	1.121 a
	50% MW Stems	278.7 ab	334.8 bc	1867.8 bc	6.37 a	1.153 a
	100% Stems	252.a b	386.6 b	2107.0 b	5.30 b	0.958 b
	100% MW Stems	305.7 a	478.9 a	2416.6 a	5.73 b	1.051 ab
	p-value	0.0589	**<0.0001**	**<0.0001**	**0.004**	0.1353
Syrah	Control	128.1 ab	62.3 d	704.5 d	4.25 a	0.747 ab
	50% Stems	74.1 b	286.1 c	1081.5 c	4.67 a	0.781 a
	50% MW Stems	130.2 ab	214.9 c	1035.4 c	3.45 a	0.630 b
	100% Stems	149.4 a	523.5 a	2086.5 a	3.58 a	0.723 ab
	100% MW Stems	138.0 ab	383.4 b	1415.5 b	3.39 a	0.607 b
	p-value	0.2001	**<0.0001**	**<0.0001**	0.4663	0.0972
**Two-way ANOVA**						
Winemaking treatment	Control	194.8 ab	99.7 c	963.4 d	5.26 ab	0.896 ab
	50% Stems	168.4 b	299.1 b	1410.9 c	5.50 a	0.951 a
	50% MW Stems	204.5 ab	274.9 b	1451.6 c	4.91 ab	0.892 ab
	100% Stems	200.7 ab	455.0 a	2096.7 a	4.44 b	0.841 b
	100% MW Stems	221.8 a	431.2 a	1916.1 b	4.56 b	0.829 b
	p-value	0.0745	**<0.0001**	**<0.0001**	0.0968	0.1277
**Main effects and interactions**						
Cultivar	*p*-value	**<0.0001**	**0.029**	**<0.0001**	**<0.0001**	**<0.0001**
Cultivar × Winemaking	*p*-value	0.1687	**<0.0001**	**<0.0001**	0.5785	0.0522

(*) Different letters within a column for each variety indicate significant differences for Fisher's LSD test and $p < 0.05$. Significant *p*-values are shown in bold fonts. Mv-3-G.: Malvidin-3-glucoside equivalents; AU: Absorbance units; CE: Catechin-equivalents.

Supplemental Table S4. Three-way analysis of variance (ANOVA) separating the effects of cultivar, winemaking treatment, and time (days after crush), of the detailed anthocyanin composition (mg/L), of Cabernet Sauvignon, Merlot and Syrah wines from the 2017 vintage. Values represent the mean of three tank replicates (n = 3).

ANOVA factor	Winemaking treatment	Total anthocyanins	Monoglucosilated anthocyanins	Acylated anthocyanins	Anthocyanin-derived pigments
Cultivar	Cabernet Sauvignon	355.81 b(*)	235.73 b	115.25 c	5.71 a
	Merlot	332.91 c	205.28 c	124.91 b	2.72 b
	Syrah	401.61 a	268.24 a	130.68 a	2.68 b
	p-value	**<0.0001**	**<0.0001**	**<0.0001**	**<0.0001**
Winemaking treatment	Control	385.84 b	248.82 b	132.44 b	4.86 a
	100% Stems	308.90 d	201.22 d	104.98 d	2.98 c
	100% MW Stems	328.52 c	214.65 c	110.87 c	3.22 bc
	Must MW	430.51 a	280.97 a	146.17 a	3.76 b
	p-value	**<0.0001**	**<0.0001**	**<0.0001**	**<0.0001**
Days after crush	1	335.02 c	215.54 c	119.48 c	0.00 c
	5	525.26 a	339.95 a	184.69 a	0.62 c
	12	437.49 b	288.17 b	147.99 b	2.50 b
	1235	156.01 d	102.01 d	42.30 d	11.70 a
	p-value	**<0.0001**	**<0.0001**	**<0.0001**	**<0.0001**
**Interactions (*p*-value)**					
cv. × W		**<0.0001**	**<0.0001**	**<0.0001**	0.110
cv. × DAC		**<0.0001**	**<0.0001**	**<0.0001**	**<0.0001**
W × DAC		**<0.0001**	**<0.0001**	**<0.0001**	**<0.0001**
cv. × W × DAC		0.089	0.031	0.241	0.078

(*) Different letters within a column for each variety indicate significant differences for Fisher's LSD test and $p < 0.05$. Significant *p*-values are shown in bold fonts.

Supplemental Table S5. Three-way analysis of variance (ANOVA) separating the effects of cultivar, winemaking treatment, and time (days after crush), of the detailed flavonol composition (mg/L), of Cabernet Sauvignon, Merlot and Syrah wines from the 2017 vintage. Values represent the mean of three tank replicates (n = 3).

ANOVA factor	Winemaking treatment	Total flavonols	Quercetin derivatives	Other flavonols	Flavonol aglycones
Cultivar	Cabernet Sauvignon	35.16 c(*)	8.50 c	22.68 b	3.98 b
	Merlot	53.56 b	26.86 a	23.57 b	3.13 b
	Syrah	64.91 a	19.44 b	28.96 a	16.51 a
	p-value	**<0.0001**	**<0.0001**	**<0.0001**	**<0.0001**
Winemaking treatment	Control	40.46 c	14.95 c	23.01 bc	2.50 c
	100% Stems	48.41 b	17.62 b	22.30 c	8.50 b
	100% MW Stems	51.63 b	18.15 b	24.64 b	8.84 b
	Must MW	64.34 a	22.36 a	30.33 a	11.65 a
	p-value	**<0.0001**	**<0.0001**	**<0.0001**	**<0.0001**
Days after crush	1	16.60 c	12.12 c	4.49 d	0.00 b
	5	59.46 b	22.85 b	36.61 b	0.00 b
	12	71.94 a	27.91 a	44.03 a	0.00 b
	1235	56.83 b	10.19 d	15.15 c	31.49 a
	p-value	**<0.0001**	**<0.0001**	**<0.0001**	**<0.0001**
**Interactions (*p*-value)**					
cv. × W		**<0.0001**	**<0.0001**	**<0.0001**	**<0.0001**
cv. × DAC		**<0.0001**	**<0.0001**	**<0.0001**	**<0.0001**
W × DAC		**<0.0001**	**<0.0001**	**<0.0001**	**<0.0001**
cv. × W × DAC		**<0.0001**	**<0.0001**	**<0.0001**	**<0.0001**

(*) Different letters within a column for each variety indicate significant differences for Fisher's LSD test and $p < 0.05$. Significant *p*-values are shown in bold fonts.
